# The role of local and remote amino acid substitutions for optimizing fluorescence in bacteriophytochromes: A case study on iRFP

**DOI:** 10.1038/srep28444

**Published:** 2016-06-22

**Authors:** David Buhrke, Francisco Velazquez Escobar, Luisa Sauthof, Svea Wilkening, Nico Herder, Neslihan N. Tavraz, Mario Willoweit, Anke Keidel, Tillmann Utesch, Maria-Andrea Mroginski, Franz-Josef Schmitt, Peter Hildebrandt, Thomas Friedrich

**Affiliations:** 1 Technische Universität Berlin, Institut für Chemie, Sekr. PC14, Straβe des 17. Juni 135, D-10623 Berlin, Germany

## Abstract

Bacteriophytochromes are promising tools for tissue microscopy and imaging due to their fluorescence in the near-infrared region. These applications require optimization of the originally low fluorescence quantum yields via genetic engineering. Factors that favour fluorescence over other non-radiative excited state decay channels are yet poorly understood. In this work we employed resonance Raman and fluorescence spectroscopy to analyse the consequences of multiple amino acid substitutions on fluorescence of the iRFP713 benchmark protein. Two groups of mutations distinguishing iRFP from its precursor, the PAS-GAF domain of the bacteriophytochrome P2 from *Rhodopseudomonas palustris*, have qualitatively different effects on the biliverdin cofactor, which exists in a fluorescent (state II) and a non-fluorescent conformer (state I). Substitution of three critical amino acids in the chromophore binding pocket increases the intrinsic fluorescence quantum yield of state II from 1.7 to 5.0% due to slight structural changes of the tetrapyrrole chromophore. Whereas these changes are accompanied by an enrichment of state II from ~40 to ~50%, a major shift to ~88% is achieved by remote amino acid substitutions. Additionally, an increase of the intrinsic fluorescence quantum yield of this conformer by ~34% is achieved. The present results have important implications for future design strategies of biofluorophores.

The development of fluorescence microscopy has opened novel possibilities for monitoring biochemical processes in cellular systems^1^,^2^,^3^,^4^,^5^. The availability of genetically encoded fluorescent proteins including photoswitchable variants provided new insights into the organization of living cells on the nanoscale by super-resolution fluorescence microscopy^6^,^7^,^8^,^9^. These techniques have a strong impact on fundamental research and are important diagnostic tools in medical science. In particular for high resolution imaging genetically encoded rather than synthetic fluorescence markers are preferred, because they can be expressed directly in the target cell and fused to the desired protein.

Fluorescent proteins emitting in the red or near-infrared spectral region gain increasing importance because they ensure a high penetration depth in tissues. Genetic engineering of the green fluorescent protein (GFP)^10^ and its homologues from other species afforded numerous variants with emission maxima covering nearly the entire visible spectrum^2^. However, red-emitting biofluorophores suffer from limited photostability and low brightness^11^. Bacterial phytochromes may overcome these drawbacks, since the tetrapyrrole cofactor exhibits a strong electronic transition between 660 and 700 nm. Furthermore, biliverdin (BV) is ubiquitous in mammalian cells as an intermediate of the heme degradation pathway. Due to their function as sensory photoreceptors, bacteriophytochromes exhibit only a low fluorescence quantum yield (Ф_fl_, ~1%) but instead undergo a photoisomerisation upon electronic excitation. However, the approach to raise Ф_fl_ by blocking the photocycle is not necessarily straightforward, since the photochemical conversion for BV-binding phytochromes of less than 10%^12^ is still low compared to the non-radiative excited-state decay pathways. Nevertheless, a bacteriophytochrome variant with strongly reduced photochemical quantum yield, that is for instance achieved by substituting the highly conserved Asp-202 (amino acid [AA] numbering refers to *Rhodopseudomonas palustris* bacteriophytochrome photoreceptor 2, RpBphP2)^13^, served as a starting point for the development of bacteriophytochromes with improved fluorescence by using evolutionary mutagenesis. Promising results were obtained by genetic engineering of the chromophore-binding domain of RpBphP2, which produced an efficient phytofluor denoted iRFP713^11^,^14^ (termed iRFP for brevity herein). This variant differs from the truncated wild-type (WT) protein RpBphP2 (including only the GAF and PAS domains, termed P2PG in the following) by a total of 13 mutations. These substitutions resulted in a Ф_fl_ of 5.9% compared to 0.7% for P2PG. Meanwhile, similar engineering strategies, also based on other bacteriophytochromes, have afforded variants with further improved fluorescence properties^14^,^15^,^16^, and the application as sensitive fluorescence probes for *in vivo* imaging has been demonstrated for some variants including iRFP^11^,^17^.

In parallel, attempts have been made to elucidate the structural basis for the altered photophysical properties in fluorescent bacteriophytochromes. Crystallographic and spectroscopic studies have consistently shown that an increased rigidity of the chromophore embedment in the protein matrix is one of the key parameters that favours fluorescence as the decay route of the electronically excited state^15^,^16^,^18^,^19^,^20^,^21^.

In this work, we continued our spectroscopic studies on iRFP^18^ to specifically analyze the contributions of individual AA substitutions on the ground- and excited-state properties of the BV cofactor. We focused on three highly conserved AAs in the chromophore-binding pocket (CBP), Asp202, Ile203, and Tyr258, which in iRFP are replaced by Thr, Val, and Phe, respectively. Mutagenesis followed two main routes via stepwise substitutions (route A) in the truncated WT P2PG and (route B) the corresponding back substitutions in iRFP (Fig. 1). The variants along route A include single, double (with two out of three possible combinations), and triple mutations. Each of the variants obtained by the corresponding back substitutions along route B also included the additional 10 substitutions of iRFP that are more remote from the CBP. The objective was to correlate chromophore structural changes determined by resonance Raman (RR) spectroscopy with the properties of the static and time-resolved fluorescence of the individual variants. The results demonstrate the coexistence of a fluorescent and a non-fluorescent conformer. The intrinsic fluorescence quantum yields for the former and its relative population are affected by both, the AA substitutions in the CBP and the remote mutations, albeit in a qualitatively different manner. The findings have implications for optimizing strategies towards generating highly fluorescent bacteriophytochromes.

## Results

### Absorption and Fluorescence Properties

In general, the electronic absorption spectra of the Pr state of all investigated P2PG and iRFP variants show very similar characteristics of the Q and Soret bands (see Supplementary Fig. S1), with variations in the Q band absorption maxima from 707 to 692 nm (Table 1). Among the P2PG-derived variants, mutations D202T and Y258F and their combination in D202T/Y258F had only a small impact on the absorption maximum, whereas the double D202T/I203V and triple D202T/I203V/Y258F mutation displayed a blue-shift by 3 and 6 nm, respectively. In a similar way, iRFP-T202D and others from route B including mutant V203I, showed markedly red-shifted absorption maxima compared to iRFP (Table 1).

The Ф_fl_ values of all investigated mutants were between those of native P2PG (0.7%) and iRFP (5.9%) (Tables 1 and 2, Supplementary Fig. S2). Interestingly, an increased Ф_fl_ seems to be accompanied with a blue-shift in the Q-band absorption maximum and an increasing Stokes shift (Table 1, Supplementary Figs S1 and S2).

None of the variants studied in this work can undergo a phototransformation to the Pfr state, but some are arrested at the Meta-R state as typically observed for phytochrome variants with substitutions of highly conserved AAs close to the chromophore^22^,^23^. Since the Qband transition of the Meta-R state exhibits reduced oscillator strength and its maximum nearly coincides with that of the parent state (Supplementary Fig. S1), IR difference spectroscopy is more reliable to detect even low photoconversion than UV-Vis absorption spectroscopy (Supplementary Fig. S5). Except for the triple mutant P2PG-D202T/I203V/Y258F, all variants generated from P2PG via route A are capable to undergo photoisomerisation to a small extent (Table 1, Supplementary Fig. S1). This observation suggests that the triple mutation D202T/I203V/Y258F represents a minimal set to completely inhibit photoconversion of P2PG, although Ф_fl_ is still relatively low. The reverse mutations starting from iRFP along route B represent a mirror image of this tendency, since the triple substitution T202D/V203I/F258Y in the CBP of iRFP is sufficient to recover photoactivity, irrespective of the 10 remote substitutions. However, blocking photoconversion alone is insufficient to optimize Ф_fl_, since the 10 remote substitutions still exhibit a profound effect: Compared to the P2PG triple mutant D202T/I203V/Y258F, Ф_fl_ increases ~2.4-fold upon introduction of the additional 10 remote substitutions in iRFP. Conversely, the iRFP triple mutant T202D/V203I/F258Y, which comprises only the 10 remote substitutions, still has a more than 2-fold larger Ф_fl_ than P2PG.

### Fluorescence dynamics

The fluorescence decays of all variants could consistently be approximated by three exponential decay components with distinct spectral dependence of the resulting decay-associated spectra (DAS, Fig. 2). The longest fluorescence lifetime was found to decrease along mutational route B from 910 ps in iRFP (Fig. 2a) to 440 ps in iRFP-T202D/V203I/F258Y (Fig. 2f). In addition, a short fluorescence decay time exists, which shortens from 350 ps in iRFP to 180 ps in iRFP-T202D/V203I/F258Y. A third component with ~100 ps present in iRFP and all route B samples without significant variations, exhibits a negative amplitude for iRFP and iRFP-F258Y in the whole spectral range (Fig. 2a,b, black curves). Such exclusively negative components in DAS are assigned to fluorescence rise effects in time^24^, representing population processes of excited states that occur in the 100 ps time regime. Since this value is close to the resolution limit of the employed TWCSPC setup, this component might be even faster than 100 ps.

Considering that decay and rise components have similar spectral characteristics, iRFP exhibits a rather homogenous excited state (Fig. 2a) with two decay components that possibly carry (phonon) sidebands at 715 and 725 nm. Such biexponential excited-state relaxations are typical for pigment-protein-complexes and do not necessarily indicate different chromophore configurations^24^. However, the iRFP-F258Y mutant already shows a heterogeneous spectral distribution of both decay components with reduced lifetimes (Fig. 2b). This feature indicates a substructure of the ground- and/or excited-state potential surface that is, in the simplest case, described by a double-well potential^24^. However, since the 90 ps component does not exhibit a transition from positive to negative amplitude (*vide infra*), it cannot be attributed to a transition between two states within the lifetime of the excited state. The two spectrally distinguishable decay components might simply represent two non-coupled excited-state subpopulations.

DAS heterogeneity is even more pronounced in iRFP-T202D and iRFP-T202D/V203I (Fig. 2c,e). Here, the fastest component (130 ps) exhibits a transition from positive values (up to about 700 nm) to negative values above 710 nm representing a novel feature not observed in iRFP and iRFP-F258Y: During the excited-state lifetime, a red-shifted emitting state is populated at the expense of a blue-shifted one. This biphasic behaviour suggests an interconversion of two chromophore configurations in the excited state. The subsequent fluorescence decay occurs with 290 ps (705 nm), and 680 ps (715 nm) (Fig. 2c). In iRFP-T202D/F258Y (Fig. 2d), the DAS heterogeneity is reduced compared to iRFP-T202D, although the emission spectrum of its longest decay component shows a more profound shoulder at 740 nm compared to iRFP. Notably, the biphasic nature of the 70 ps component is absent suggesting that the effects of both mutations on the DAS partially neutralize each other.

A further dissection into several spectrally distinguishable states is observed in the triple mutant iRFP-T202D/V203I/F258Y, still carrying all remote substitutions of iRFP. Besides a remarkable acceleration of all fluorescence decay components, which accounts for the small Ф_fl_, the DAS of this mutant distinguishes at least four spectral bands at ~695, ~705, ~715 and ~730 nm (Fig. 2f), indicating radiative decays from four distinct excited states or chromophore configurations. The pronounced biphasic nature of the 100 ps component indicates strong coupling and interconversion between the electronic states at 695 nm and at 715 nm, followed by a ~440 ps decay. Thus, already single back-substitutions in the CBP of iRFP entail substantial excited-state heterogeneity, which gradually increases with the number of mutations.

Notably, the DAS of iRFP-T202D/V203I/F258Y is similar to that of the parental P2PG (Fig. 2g), which also exhibits a biphasic component (180 ps) and two further decay components (280 ps and 690 ps). Among them, the faster one dominates in amplitude, in line with the lowest Ф_fl_ of P2PG. In total, four spectral features at 710, 730, 750, and 780 nm can be discriminated for P2PG. In contrast, the double mutant P2PG-D202T/F258Y, which only carries two CBP substitutions, already shows rather homogenous DAS (Fig. 2h), essentially similar to iRFP, despite the fact that the mutant still shows photoconversion. Figure 3a summarizes the average lifetimes and Fig. 3b compares the fast and slow fluorescence decay time constants of the constructs from Fig. 2.

### Resonance Raman Spectroscopy

All phytochrome variants studied in this work were in the Pr state as reflected by the characteristic vibrational band pattern of the chromophore in the *ZZZssa* configuration (see Supplementary Information). For a detailed vibrational assignment we therefore refer to previous analyses^25^,^26^. In this work we focus on the identification of selected modes that correlate with specific structural parameters of the tetrapyrrole. Between 1565 and 1580 cm^−1^, the protonation marker band of the Pr state is observed^27^,^28^. It is due to the in-phase N-H in-plane bending (N-H ip) of the ring *B* and *C* N-H groups and thus indicates that all pyrrole nitrogen atoms carry a proton and rings *B* and *C* share a positive charge (Figs 4 and 5). In P2PG and iRFP, this band is observed between 1571 and 1575 cm^−1^, and it shifts down to 1075 and 1079 cm^–1^ in D_2_O^18^, indicating a cationic (protonated) chromophore in each case. The same conclusion can be drawn for all P2PG or iRFP variants. Small frequency variations observed for the N-H ip indicate minor changes of the hydrogen bond interactions of the ring *B* and *C* N-H groups^25^.

The most intense peak in the Pr state of all variants is observed around 1620 cm^−1^ (Figs 4 and 5, left). It is actually composed of two overlapping bands, which are more clearly discriminated in the second-derivatives (grey traces in Figs 4 and 5). The underlying modes include mainly the C=C stretching coordinates of the *C*-*D* methine bridge (*C*-*D* stretching) and of ring *D* and its vinyl substituent, albeit with different relative contributions. Since the *C*-*D* stretching couples with the N-H ip of rings *C* and *D*, the main character of the two modes can be distinguished on the basis of the H/D isotopic shifts (Figs 4 and 5). In all cases, the frequency downshifts of the band components at 1620 and 1627 cm^−1^ is about 7 and 2 cm^−1^, respectively. Thus, the lower and higher frequency components may be considered as *C*-*D* stretching and ring *D* C=C stretching modes, respectively.

The corresponding C=C stretching mode of the *A*-*B* methine bridge (*A*-*B* stretching) is observed between 1640 and 1660 cm^−1^. In the Pr state of many BV-binding phytochromes including P2PG, this mode is split into two components, corresponding to two conformational sub-states. We denote the states represented by the low- and high-frequency component as state I and II, respectively. For P2PG these bands are found at 1641 and 1651 cm^−1^ (Fig. 4, left). Due to the coupling of the *A*-*B* stretching with the N-H ip coordinates of rings *A* and *B*, these modes shift down by ~10 cm^−1^ upon D_2_O exchange. Thus, only one of these modes can be safely detected in the RR spectra measured in D_2_O (i.e., 1641 cm^−1^; Fig. 4, right), whereas the lower-frequency component overlaps with the (largely H/D-insensitve) C=C stretching mode of ring *D*.

Among the modes in the region between 1550 and 1670 cm^−1^, the *A*-*B* stretching and the ring *D* C=C stretching display the most notable differences between P2PG and iRFP. First, the two *A*-*B* stretching modes are of nearly equal intensity in P2PG, but the intensity of the high frequency component strongly increases in iRFP along with a frequency upshift from 1651 to 1656 cm^−1^. Inspection of the protein variants of route A (Fig. 4) indicates that the single substitution of either Asp202 or Tyr258 (D202T, Y258F) already causes this frequency upshift while the intensity ratio *I*_*II*_*/I*_*I*_ (=*R*; Table 1) of the two *A-B* stretching modes varies only slightly by the individual AA replacements in the CBP. However, the 10 remote substitutions that afford the iRFP variant cause a major intensity redistribution of the two bands. The latter effect appears to be independent of the CBP substitutions since all variants from route B, each involving the 10 remote substitutions, display very high *I*_*II*_*/I*_*I*_ intensity ratios of the two *A*-*B* stretching modes (Fig. 5). Note that the low-frequency component at 1641 cm^−1^ in P2PG also shifts up to higher frequencies upon substitutions in the CBP (1646 cm^−1^ in P2PG-D202T/I203V/Y258F) but the additional remote substitutions revert this shift and keep the band position at 1642 cm^−1^ (Figs 4 and 5; Table 1).

Also for the ring *D* C=C stretching, the frequency upshift from P2PG (1625 cm^−1^) to iRFP (1629 cm^−1^) is already complete in P2PG-D202T/I203V/Y258F, but not fully reversed in P2PG-D202T/I203V/Y258F. Further spectral data reflecting mutation-induced structural changes of the chromophore including the *C*-*D* methine bridges are provided in the Supporting Information (Supplementary Figs S3 and S4).

## Discussion

Phytochrome variants that display enhanced fluorescence quantum yield include two groups of AA substitutions. The first group refers to positions in the immediate environment of the chromophore (CBP substitutions). These are specifically D202, I203, and Y258 which all have contacts with the BV cofactor or with the surrounding H-bond network. In P2PG, substitutions at these positions account for a Ф_fl_ increase from 0.7 to 2.5%. The further increase to 5.9% is only achieved by including a second group of 10 substitutions remote from the CBP.

The impact of substituting D202 and Y258 on the structure of the chromophore pocket has been recently analysed for the Pr state of the chromophore-binding domain CBD-DR^15^ of the *Deinococcus radiodurans* bacteriophytochrome (AA numbering refers to P2PG). Crystal structures revealed a perturbation of the hydrogen bond network in the chromophore pocket, particularly affecting the interactions with the ring *A* carbonyl. This was suggested to impair excited-state proton transfer that competes with the radiative excited-state decay. As an additional factor responsible for the about 2-fold increased fluorescence in the D202H and Y258F single and D202H/Y258F double mutants, the *E* configuration at the *C*-*D* methine bridge was proposed to be destabilized, corresponding to a decrease of the photochemical quantum yield. Indeed, the structural changes of the chromophore refer to the *A*-*B* and *C*-*D* methine bridges. Specifically, the mutations cause an increase of the dihedral angle C(4)-C(5)-C(6)-N(*B*) from 6.7° (WT CBD-DR) to 10.4° and 14.0° in the D202H and D202H/Y258F mutants, respectively^15^. The dihedral angle C(14)-C(15)-C(16)-N(*D*) of the *C*-*D* methine bridge displays the opposite tendency as it decreases from 35.8° to 0.5° and 12.6° in the D202H and D202H/Y258F mutants, respectively. This geometric change corresponds to a substantial decrease of the tilt angle of ring *D* with respect to ring *C* by ~15°.

In view of the far-reaching structural similarities between CBD-DR and P2PG, one may expect similar mutation-induced structural changes also for P2PG, although in the latter case the ring *D* tilt angle is already rather low in the WT protein^29^. In fact, the vibrational modes localized at the *A*-*B* methine bridge and in ring *D* respond to substitutions of D202 and Y258. Note that the correlation with the structural changes in the CBD-DR mutants are justified since control experiments with the D202H mutant of P2PG display the same tendency in the RR spectra as threonine substitution at this position studied in this work (Supplementary Fig. S6). The single mutants P2PG-D202T and -Y258F as well as P2PG-D202T/Y258F show essentially the same frequency upshifts of these modes compared to P2PG (Table 1), and the additional I203V substitution in the triple mutant causes only a further 1-cm^−1^ shift of the ring *D* mode. These findings may be rationalized in terms of an increased C(4)-C(5)-C(6)-N(*B*) dihedral angle at the *A*-*B* methine bridge and a reduced tilt angle of ring *D*. This conclusion is also consistent with the concomitant increase of the frequency of the *C*-*D* HOOP mode (Supplementary Figs S3 and S4) that has been shown to exhibit a negative correlation with the C(14)-C(15)-C(16)-N(*D*) dihedral angle^30^, thereby indicating a reduced torsion of the *C*-*D* methine bridge upon mutations in the CBP, particularly of either D202 or Y258. These chromophore structural changes as revealed by the RR spectra account for an increase of Ф_fl_ from 0.7% (P2PG) to 2.5% (P2PG-D202T/I203V/Y258F) (Supplementary Fig. S7), in line with the previously proposed mechanisms for promoting radiative excited state decay^15^,^19^,^21^. A decrease of the tilt angle as indicated by the frequency upshift of the ring *D* and the HOOP mode stabilizes the *Z* vs. the *E* configuration of the *C*-*D* methine bridge (*vide supra*), consistent with a complete inhibition of photoisomersiation in the P2PG triple mutant which displays the highest ring *D* stretching frequency. This remarkably critical and as yet unrecognized influence of the rather conservative I203V mutation on photoconversion is reflected by the mutants from route B, since introduction of mutation V203I into iRFP-T202D/F258Y marks the transition between constructs that do or do not undergo photoconversion. Also, the concomitantly increased torsion of the *A*-*B* methine bridge may contribute to the stabilization of *Z* configuration of the chromophore, but – according to the crystallographic analyses^15^,^16^ – it has an additional effect on the H-bond network in the CBP involving the ring *A* carbonyl. However, the latter mode is rather weak in RR and, as far as the technique was applicable, also in the IR difference spectra precluding further analyses of mutation-induced effects (Supplementary Fig. S5).

In addition to the three CBP substitutions, 10 remote replacements account for a further increase of Ф_fl_ from 2.5 to 5.9% (Table 1), which is not correlated with frequency shifts of the stretching modes (Supplementary Fig. S7). Instead, we note major intensity redistributions between the two conjugate *A*-*B* stretching modes such that the high-frequency component (conformer II) clearly dominates in the spectrum of iRFP, corresponding to a distinct decrease of the structural heterogeneity. The mole fractions of the two conformer states I and II (*x*_*I*_ and *x*_*II*_) can be approximated from the ratio *R* = *I*_*II*_*/I*_*I*_ of the relative intensities *I*_*I*_ and *I*_*II*_ of the low- and high-frequency component of the *A*-*B* stretching mode, yielding *R*/(1 + *R*) for the mole fraction *x*_*II*_ of conformer II, which, e.g., rises from 50% (P2P2-D202T/I203V/Y258F) to 88% in iRFP. We therefore conclude that the remote substitutions primarily affect structural packing of the protein, which strongly favours conformer II.

The simplest explanation of the present results is based on a distribution between two conformers (I, II), which solely differ by their fluorescence quantum yields Φ_*I*_ and Φ_*II*_, in analogy to a previous proposal for cyanobacterial phytochromes^31^. Then, the experimentally determined fluorescence quantum yield Φ_*fl*_ is given by


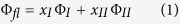


Again, *x*_*I*_ and *x*_*II*_ are the mole fractions of the two conformer states as noted above. Assuming identical Raman cross sections for the conjugate *A*-*B* stretching modes, eq. (1) can be rewritten to


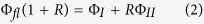


The values for *R* were evaluated from the second derivatives in Figs 4 and 5 (left, grey traces; Table 1) to obtain a plot of Φ_*fl*_(1 + *R*) vs. *R* (Fig. 6).

In fact, except for the iRFP triple mutant and the iRFP-F258Y mutant of route B (red points in Fig. 6), the data are correlated and indicate that Φ_*fl*_ increases with increasing population of conformer II. However, in contrast to the simple expectation from eq. (2), the data follow a parabolic (blue line) rather than a linear function (red line), and a linear fit would afford a physically meaningless negative intercept. These deviations from linear behaviour can be rationalized since eq. (2) assumes that (i) the fluorescence quantum yields of each conformer remain unchanged in the individual protein variants, and (ii) the variations of the experimentally determined Ф_*fl*_ solely depend on the relative populations of the two conformers. However, the increase of Ф_*fl*_ from 0.7% (P2PG) to 2.5% in the P2PG triple mutant (route A) can only partly be attributed to a slightly larger population of the “fluorescent” conformer II (~50% vs. ~40% in P2PG). Instead, the CBP substitutions perturb the chromophore structure, as reflected by the changes of the *A*-*B* and ring *D* stretching modes, and thus affect the fluorescence properties *including* the fluorescence quantum yields. Additional evidence for this conclusion is derived from the DAS.

Unlike RR spectroscopy, which samples all conformers in the ground state, time-resolved fluorescence spectroscopy monitors radiative processes occurring within the lifetime of the chromophore’s excited state. Accordingly, the DAS spectra predominantly reflect the excited-state processes of the (fluorescent) conformer II, while those of the non-fluorescent conformer I remain largely invisible. Consequently, the substantial excited-state heterogeneity reflected by the DAS of P2PG does not primarily mirror the ground-state heterogeneity of this protein with the comparable populations of the states II (~40%) and I (~60%). Instead, the DAS reveal a variety of possible dissipative decay processes for conformer II. This excited-state heterogeneity is already largely removed by mutation of the two most important residues in the CBP (D202T/Y258F), while the conformer ratio (45%/55%) remains nearly unchanged. Thus, the concomitant increase of Ф_*fl*_ by ~2 is mainly a consequence of the structural changes of conformer II, as discussed above. Following this interpretation, one can readily rationalize that iRFP is endowed with a spectrally homogenous fluorescence emission whereas the triple mutant iRFP-T202D/V203I/F258Y, which has the essential CBP residues of P2PG in place and comprises only the 10 remote mutations, shows a heterogeneous DAS closely resembling P2PG, in line with both having the same CBP residues. The Ф_*fl*_ value of 1.5% for the triple mutant is clearly higher than that of P2PG of (0.7%), which is attributed to the distinctly higher population of the fluorescent conformer II. This can be quantitatively verified by combining eq. (2) for P2PG (superscript “A”) and the triple mutant (superscript “B”) to afford Φ_*II*_ according to


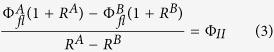


Thus, one obtains Φ_*II*_ = 1.7% and, with eq. (2), Φ_*I*_ = 0.06%, which confirms the notion of a fluorescent conformer II and a non-fluorescent conformer I.

We now assume that Φ_*I*_ ≈ 0 holds for all variants. Even if the CBP substitutions caused an increase of the intrinsic fluorescence quantum yields of both conformers by a similar factor, Φ_*I*_ would remain distinctly smaller than 1%, such that the contribution of conformer I to the experimentally determined fluorescence quantum yield can be neglected. Then eq. (2) simplifies to


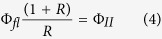


such that the intrinsic fluorescence quantum yields of conformer II can be evaluated for each mutant (Table 2).

The stepwise substitution of the three CBP residues reveal the expected steady increase of Φ_*II*_, with the strongest increase (from 1.7% to 3.2% or 3.6%, respectively) for the single substitutions of D202 and Y258 (Table 2), in line with the most pronounced changes in the DAS and RR spectra. A further increase (to 4.4%) is noted for the P2PG-D202T/Y258F double mutant, although the contributions by the individual substitutions do not act simply additive. In concert with D202T, the I203V substitution has no effect on Φ_*II*_, but contributes to the further increase of Φ_*II*_ (to 5%) in the triple mutant P2PG-D202T/I203V/Y258F.

We now compare the variants which only differ by the remote substitutions (Table 2, shaded grey). Except for P2PG and the triple mutant of route B, all other variant pairs reveal an increase of Φ_*II*_ induced by the remote substitutions, particularly pronounced (~34%) for the pair P2PG-D202T/I203V/Y258F and iRFP. These results show that the remote substitutions exert a dual function: a shift of the conformational distribution towards the fluorescent conformer II and a further increase of Φ_*II*_. The latter effect is not reflected by distinct differences in the respective RR spectra and may be due to a more rigid fixation of conformer II within the protein which is likely to reduce non-radiative excited-state decays^19^. It is interesting to note that the latter effect is not observed for the conjugate pair P2PG and iRFP-T202D/V203I/F258Y. This may be related to the fact that the fluorescence-optimized iRFP was obtained by random mutagenesis starting from the D202H mutant instead of the WT P2PG^11^.

## Conclusions

The present spectroscopic analysis revealed that the chromophore of P2PG-derived variants exists in fluorescent and non-fluorescent conformational states, probably a common feature of the Pr form of prototypical phytochromes^12^. Generating highly fluorescent phytofluors thus requires shifting the conformational distribution towards the fluorescent conformer and optimizing its structure to raise the probability for radiative excited-state decay. This increase of the intrinsic fluorescence quantum yield primarily involves structural changes at the *C*-*D* and *A*-*B* methine bridges, induced by AA substitutions in the CBP. These structural changes reduce and eventually block photoconversion and might also abolish excited-state proton transfer as a competing decay channel^15^,^19^. Whereas such structural changes in CBP and their consequences on the excited-state processes may become predictable on the basis of crystallographic, spectroscopic, and theoretical analyses, this will be more difficult for remote substitutions, which in P2PG primarily enrich the population of the fluorescent conformer, but also further increase its intrinsic fluorescence quantum yield, presumably via enhancing the rigidity of the chromophore packing, which lowers the yield for internal conversion. Since the effects particularly of critical remote substitutions are difficult to predict on the atomic level due to the limitations of current computational resources, the complexity of the optimization problem will, for quite some time, need to rely on combinatorial engineering, rather than rational design approaches.

## Materials and Methods

### Site directed mutagenesis, protein expression and purification

**Figure 1:**
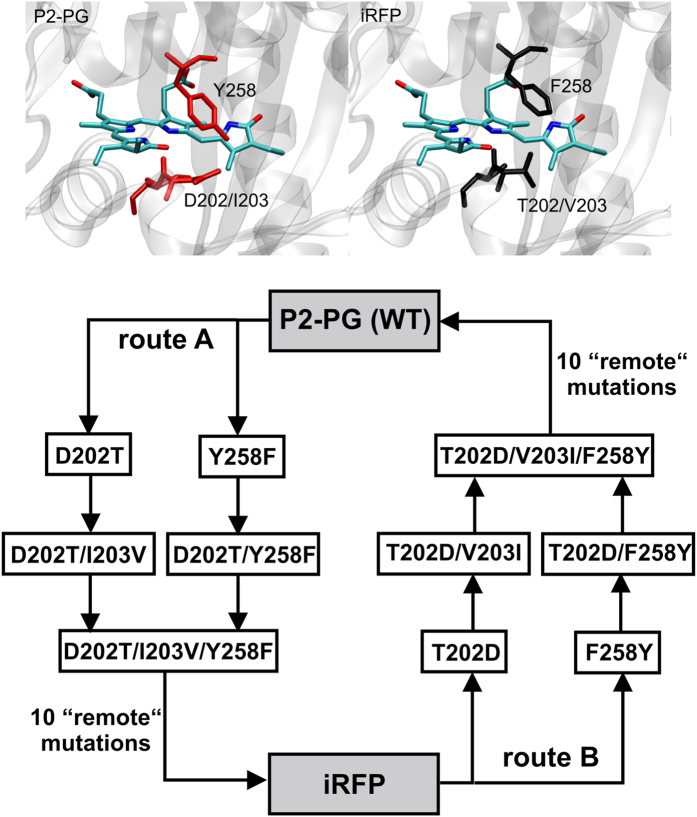
Top, structure of the chromophore binding pocket of P2PG (left) and iRFP (right), indicating the amino acid variations between both variants. The structural models^18^ were derived from the crystal structure of the chromophore binding domain of RpBphP2 obtained by homologue-directed mutagenesis, which was termed RpBphP2-CBD* 29. Bottom, schematic presentation of the step-wise amino acid substitutions starting from P2PG WT (route A) and from iRFP (route B).

**Figure 2:**
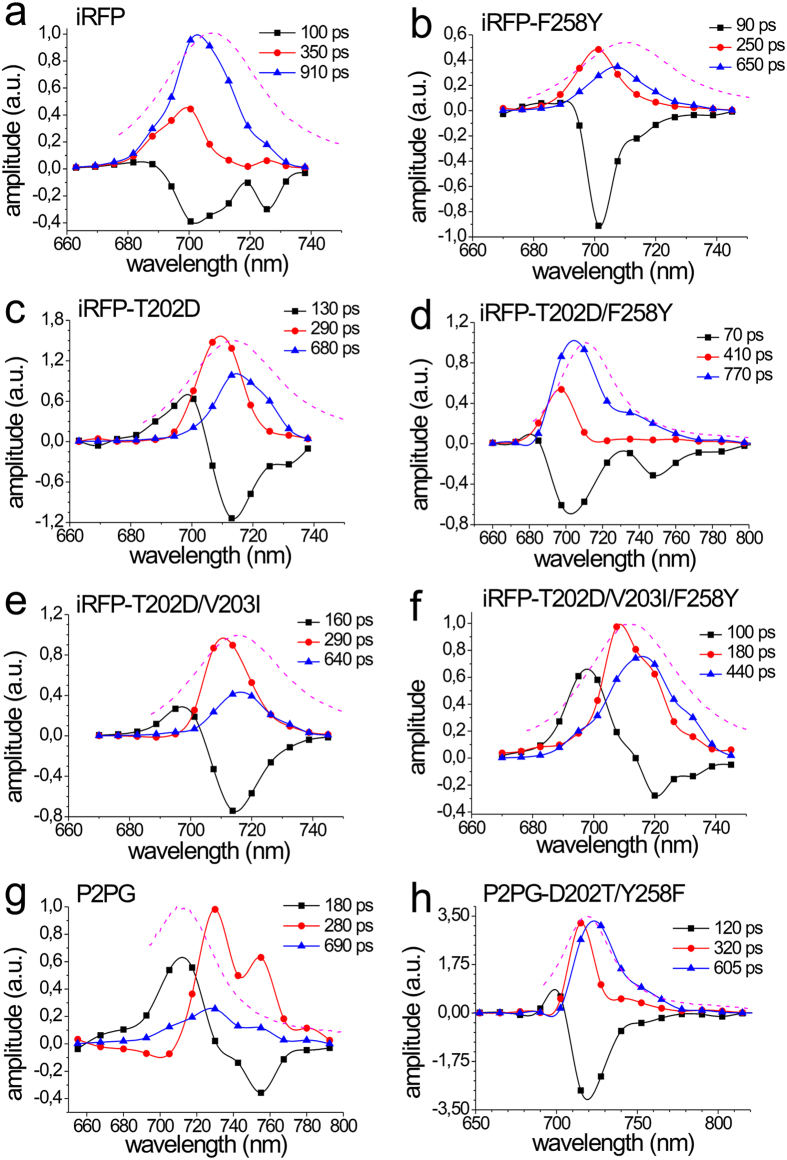
Decay associated spectra (DAS) and fluorescence lifetimes of iRFP, P2PG and selected mutants from route A and B. (**a–h**) The DAS were obtained from global fitting of the wavelength-resolved fluorescence decays recorded at 10 K with a sum of three exponentials. The relative amplitudes of the individual decay components (ultrafast: black; fast: blue; slow: red) from these fits are depicted for each wavelength channel, with time constants as given in the insets. The black, blue and red curves are included to guide the eye. Superimposed to the DAS are the corresponding fluorescence emission spectra (magenta) of the protein variants measured at room temperature.

**Figure 3:**
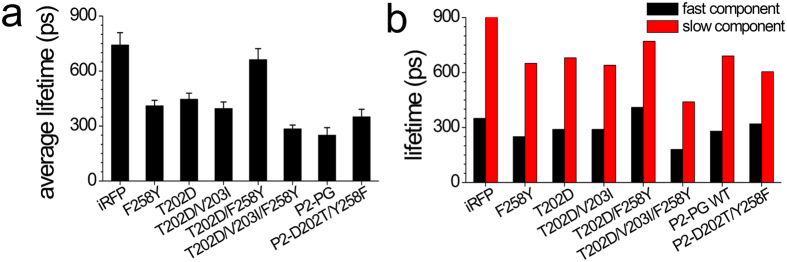
Average fluorescence lifetimes (**a**) and overview about the lifetimes (**b**) of the fast (black columns) and slow (red columns) component for each construct from Fig. 2a–h.

**Figure 4:**
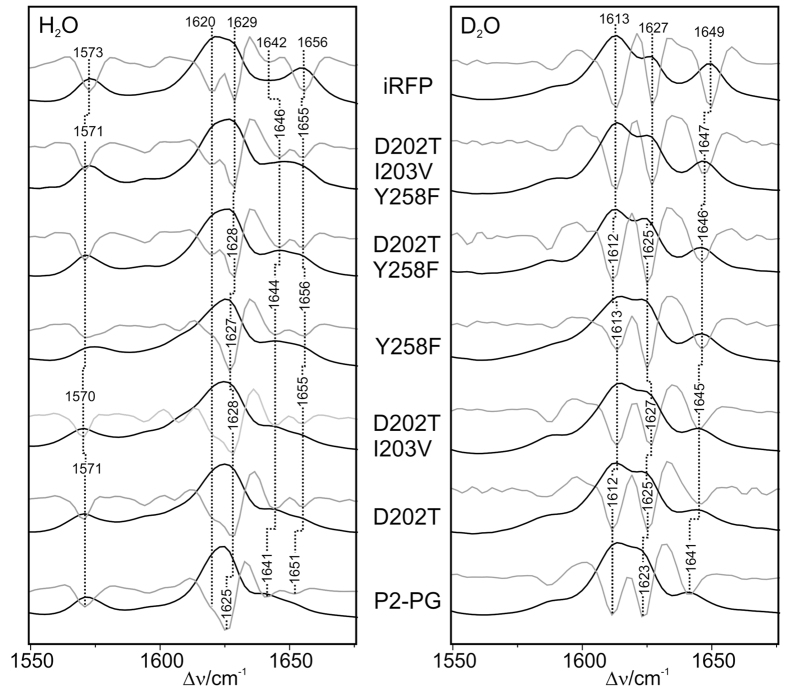
RR spectra of the P2PG variants obtained via route A (from bottom to top), compared to the spectra of P2PG WT and iRFP. The spectra, measured from the proteins in H_2_O (left) and D_2_O (right), display the region of the C=C stretching modes. Grey traces represent the second derivatives of the spectra.

**Figure 5:**
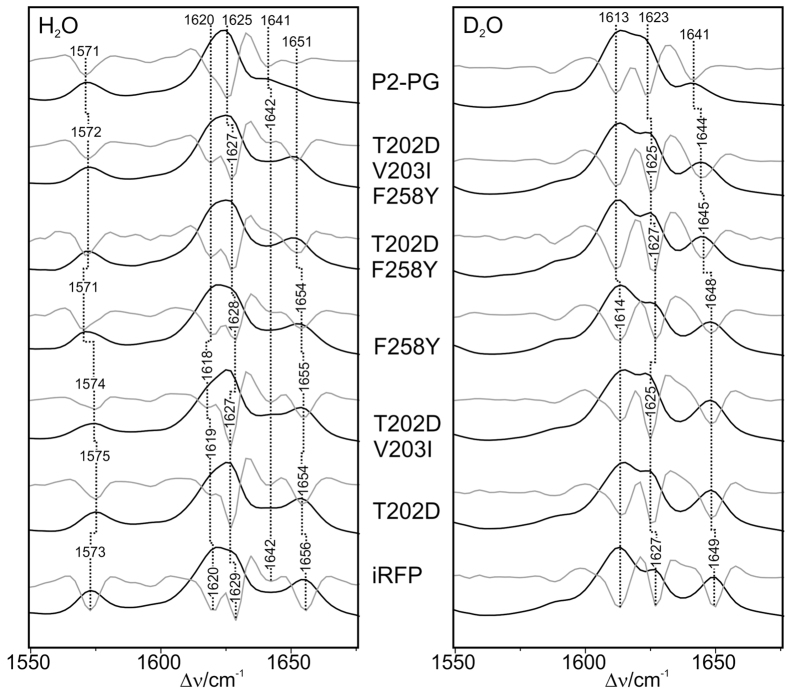
RR spectra of the P2PG variants obtained via route B (from bottom to top), compared with the spectra of P2PG WT and iRFP. The spectra, measured from the proteins in H_2_O (left) and D_2_O (right), display the region of the C=C stretching modes. Grey traces represent the second derivatives of the spectra.

**Figure 6:**
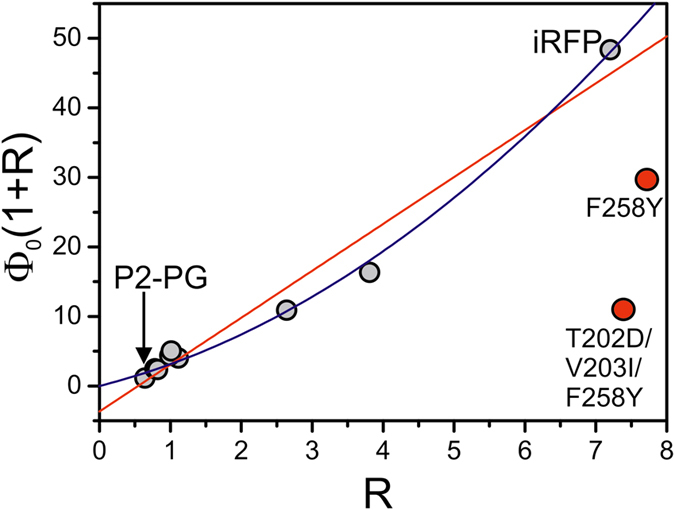
Correlation of the experimentally determined fluorescence quantum yield Φ_*fl*_ and the relative population of conformer II according to eq. (2). The red line represents a linear fit of eq. (2) to the experimental data (except for iRFP-F258Y and iRFP-T202D/V203I/F258Y). The blue curve describes a second order polynomial fit to the data to illustrate the deviation from the linear behavior.

The cDNA templates used in this study were either the plasmid pQE81L containing the cDNA of the RpBphP2 PAS-GAF domains, which was obtained by artificial gene synthesis upon codon optimization for mammalian cells (GeneArt, Regensburg, Germany), or the plasmid pQE81L containing the iRFP cDNA, as described^18^. Mutagenesis was performed using the QuikChange^®^ Site-Directed Mutagenesis Kit (Stratagene, La Jolla, USA) according to manufacturer’s instructions, which resulted in the following constructs: P2PG-Y258F, P2PG-D202T, P2PG-D202T/Y258F, P2PG-D202T/I203V, P2PG-D202T/I203V/Y258F (for mutational route A, see Fig. 1), and iRFP-T202D, iRFP-F258Y, iRFP-T202D/V203I, iRFP-T202D/F258Y iRFP-T202D/V203I/F258Y (for mutational route B, see Fig. 1). Oligonucleotides were obtained from MWG Eurofins Operon (Ebersberg, Germany) and cDNAs of all constructs were verified by sequencing (MWG Eurofins Operon). The various iRFP and P2PG construct plasmids were co-transformed in NEBturbo cells with the previously described pQE81L-Kan plasmid bearing the gene for the human heme oxygenase type 2 (hHOX2)^18^. The cells were grown overnight at 37 °C on LB-Agar plates containing 100 μg/mL ampicillin and 50 μg/mL kanamycin. The more recently mutated clones (iRFP-T202D/F258Y, P2PG-D202T/I203V, P2PG-D202T/I203V/Y258F) were transformed in DH5 alpha cells with the gene for the hHOX2 stably integrated in the genome using the method described by Kuhlman and Cox^32^. Transformed cells were grown overnight at 37 °C on LB-Agar plates containing 100 μg/mL ampicillin. Details of the protein expression and purification protocol are given elsewhere^18^. According to the SAR values, holoprotein assembly was comparable in the variants of both mutational routes (P2PG and iRFP). Purified proteins were frozen in liquid nitrogen and stored at −80 °C. For spectroscopic measurements, phytochrome samples were prepared in H_2_O or D_2_O (99.95%, Deutero GmbH) Tris buffer (50 mM Tris/Cl, 5 mM EDTA, and 300 mM NaCl). The pH (pD) was adjusted to pH = 7.8 (pD = 7.8) using a 3 M HCl (DCl, 99% in D_2_O, Sigma-Aldrich, Deisenhofen, Germany) pH electrode. Final protein concentrations were ca. 500 μM for RR and IR experiments, but distinctly lower for fluorescence measurements (*vide infra*).

### Vibrational spectroscopy

RR spectroscopic measurements were carried out as described previously using a Fourier-transform (FT) Raman spectrometer with 1064-nm excitation^18^. All RR spectra shown in this work were measured at −140 °C. Difference IR spectroscopy measurements of photochemically active P2PG and iRFP derivatives were performed at ambient temperature using an IFS28 spectrometer (Bruker) equipped with a liquid nitrogen-cooled MCT detector. Approximately 2–4 μL of protein solution were placed in a 3 μm cavity between two thin CaF_2_ windows (d = 20 mm) and sealed with silicone grease. Forward (reverse) conversion from the dark adapted state (photoproduct) was achieved by irradiation with a 660 nm (780 nm) LED array.

### Absorption and static fluorescence spectroscopy

All measurements were performed at room temperature under protective green light (502 nm). Protein samples were prepared in Tris buffer and measured in disposable cuvettes (10 mm path length). UV/VIS measurements were performed immediately prior to fluorescence measurements with a Cary 1E Varian spectrophotometer (Agilent Technologies). In the case of photochemically active variants, the presence of only the parent Pr state was ensured by LED irradiation at 780 nm. Fluorescence measurements were performed using a Fluoromax 2 spectrometer (Horiba Scientific). Excitation was set to 20 nm blue-shifted from the Q band maximum of the absorption spectra (690–700 nm; see Supplementary Fig. S1). The fluorescence signal was collected, starting from 5 nm above excitation up to 900 nm, and corrected according to the number of absorbed photons (absorbance at the excitation wavelength), using the molar extinction coefficient of iRFP (85,000 M^−1^ cm^−1^)^18^ as a reference. Standard solutions of the reference dyes Atto 680 in H_2_O (Attotech) and Nile Blue (Sigma Aldrich) in ethanol (Ф_fl_ = 0.3/0.27) were used to determine the iRFP quantum yield (Ф_fl_ = 0.059). Subsequently, iRFP was then used as a reference for determining the fluorescence quantum yields of the other phytochrome variants.

### Time- and wavelength-correlated single photon counting (TWCSPC)

Measurements were performed with the setup as described^33^. Cooling of the measurement cuvette down to 10 K was performed using a metal alloy cuvette holder connected to a home-built variable-temperature cryostat (10–300 K, CTI-Cryogenics 8001/8300) equipped with a thermocouple directly connected to the cooling head for temperature control. In the case of photochemically active variants, in particular P2PG WT, the presence of only the parental Pr state was ensured by continuous LED irradiation at 780 nm during freezing. For TWCSPC, a Hamamatsu R5900 16-channel multi-anode photomultiplier tube (PML-16C, Becker & Hickl, Berlin, Germany) was employed and signals were registered with a SPC-130 (Becker&Hickl) measurement card. A polychromator with a 1200 grooves/mm grating ensured a spectral bandwith of 6.25 nm per channel. A 632 nm pulsed laser diode (BHL-635, Becker&Hickl, Berlin) was used for excitation. The time- and wavelength-resolved fluorescence spectra were analysed by global fits using three exponentials for all decay curves measured in one spectrum affording common values of lifetimes *τ*_*j*_ (linked parameters) for all decay curves and wavelength-dependent pre-exponential factors *a*_*j*_(*λ*) (non-linked parameters), thus yielding the decay-associated spectra (DAS) of the individual decay components. The quality of the fit was judged by the value of χ_r_^2^ and by the degree of randomness of residuals to check for the absence of any correlation of the deviations in a certain time interval (for further details, see Supporting Information).

## Additional Information

**How to cite this article**: Buhrke, D. *et al*. The role of local and remote amino acid substitutions for optimizing fluorescence in bacteriophytochromes: A case study on iRFP. *Sci. Rep.* **6**, 28444; doi: 10.1038/srep28444 (2016).

## Supplementary Material

Supplementary Information

## Figures and Tables

**Table 1:** Absorption and fluorescence maxima, and fluorescence quantum yields of various P2-PG variants.

protein variant	absorption (nm)	fluorescence (nm)	Φ_fl_ (%)	photo- activity	ring *D* str. (cm^−1^)	*A-B* str. (cm^−1^)		Intensity ratio *A-B* str., conformer II/conformer I (*R* = *I*_*II*_*/I*_*I*_)
						conformer I	conformer II	
P2-PG variants derived from P2-PG WT (route A)								
WT P2-PG	707	712	0.7	yes	1625	1651	1641	0.64
D202T	707	717	1.4	yes	1628	1655	1644	0.78
Y258F	706	717	1.9	very weak	1627	1656	1644	1.11
D202T/I203V	704	716	1.3	yes	1628	1655	1644	0.82
D202T/Y258F	707	719	2.2	very weak	1628	1655	1646	0.99
D202T/I203V/Y258F	701	715	2.5	no	1629	1655	1646	1.01
P2-PG variants derived from iRFP (route B)								
iRFP	692	708	5.9	no	1629	1656	1642	7.20
T202D	697	714	3.4	no	1627	1654	1642	3.81
F258Y	693	710	3.6	no	1628	1654	1642	7.22
T202D/V203I	702	716	3.0	no	1627	1655	1642	2.64
T202D/F258Y	697	710	2.8	no	1627	1652	1642	n.a.
T202D/V203I/F258Y	699	712	1.5	yes	1627	1652	1642	7.38

The variants (route A and B) are defined in Fig. 1. Absorption and fluorescence maxima were taken from Supplementary Figs S1 and S2 (Supporting Information); the fluorescence quantum yields (Φ_fl_ in %) were determined experimentally (see materials and methods). The stretching mode frequencies of the *A*-*B* methine bridge and of ring *D* as well as the intensity ratio *R* (intensity of the high [conformer II] vs. the low frequency component [conformer I] of the *A*-*B* mode) were determined from second derivatives of the RR spectra in Figs 5 and 6 (left panels). For the iRFP-T202D/F258Y variant, the error in the intensity determination was too large due to a close overlap of the respective bands (Fig. 5, left) and a very low intensity of the conformer I component (thus denoted as “n.a.” = not applicable).

**Table 2:** Intrinsic fluorescence quantum yields of conformer II.

Variants without remote substitutions			Variants with remote substitutions			Intrinsic fluorescence quantum yields Φ_*II*_	
Route A variants	Φ_fl_ (%)	R	Route B variants	Φ_fl_	R	Φ_*II*_(A) (%)	Φ_*II*_(B) (%)
P2-PG	0.7	0.64	T202D/V203I/F258Y	1.5	7.38	1.7	1.7
D202T	1.4	0.78	—	—	—	3.2	—
Y258F	1.9	1.11	T202D/V203I	3.0	2.64	3.6	4.1
D202T/I203V	1.3	0.82	F258Y	3.6	7.72	2.9	4.1
D202T/Y258F	2.2	0.99	—	—	—	4.4	—
D202T/I203V/Y258F	2.5	1.01	iRFP	5.9	7.20	5.0	6.7
—	—	—	T202D	3.4	3.81	—	4.3
—	—	—	T202D/F258Y	2.8	n.a.	—	—

The intrinsic fluorescence quantum yields Φ_*II*_ were evaluated according to eq. (4) using the experimentally observed fluorescence quantum yields Φ_fl_ and the intensity ratio *R* (see Table 1), taken to be equal to the population ratio of the conformers II and I. Φ_*II*_(*A*) and Φ_*II*_(*B*) refer to values determined for the variants of route A and B, given in the same row of the table. For the iRFP-T202D/F258Y variant, the error in the intensity determination was too large due to a close overlap of the respective bands (Fig. 5, left) and a very low intensity of the conformer I component (thus denoted as “n.a.” = not applicable).
