# Supplementary material for: The role of local and remote amino acid substitutions for optimizing fluorescence in bacteriophytochromes: A case study on iRFP
Source: Sci Rep. 2016 Jun 22;6:28444. doi: 10.1038/srep28444 (PMC4916461; doi:10.1038/srep28444)
Supplement: Supplementary Information [file srep28444-s1.pdf]

Supporting information

The role of local and remote amino acid substitutions for optimizing fluorescence in bacteriophytochromes: A case study on iRFP

David Buhrke, Francisco Velazquez Escobar, Luisa Sauthof, Svea Wilkening, Nico Herder, Neslihan N. Tavraz, Mario Willoweit, Anke Keidel, Tillmann Utesch, Maria-Andrea Mroginski, Franz-Josef Schmitt, Peter Hildebrandt*, and Thomas Friedrich*

Technische Universität Berlin, Institut für Chemie, Sekr. PC 14, Straße des 17. Juni 135, D-10623 Berlin, Germany

***Corresponding authors:** Thomas Friedrich, Peter Hildebrandt, Technische Universität

Berlin, Institut f. Chemie, Sekr. PC 14, Straße des 17. Juni 135, D-10623 Berlin, Germany,

Tel: +49-(30)-31421419, Fax: +49-(30)-31421122, Email: hildebrandt@chem.tu-berlin.de

Tel: +49-(30)-31424128, Fax: +49-(30)-31478600, E-mail: friedrich@chem.tu-berlin.de

Content:

1. **Nomenclature of chromophore configurations**
2. **Fluorescence data analysis**
3. **Absorption spectra and photoconversion (Fig. S1)**
4. **Fluorescence spectra (Fig. S2)**
5. **RR spectra in the low frequency region (Figs. S3, S4)**
6. **IR difference spectra (Fig. S5)**
7. **RR spectra of further mutants (Figs. S6)**
8. **Correlation of Raman shifts with fluorescence quantum yields (Fig. S7)**

1. Nomenclature of chromophore geometry

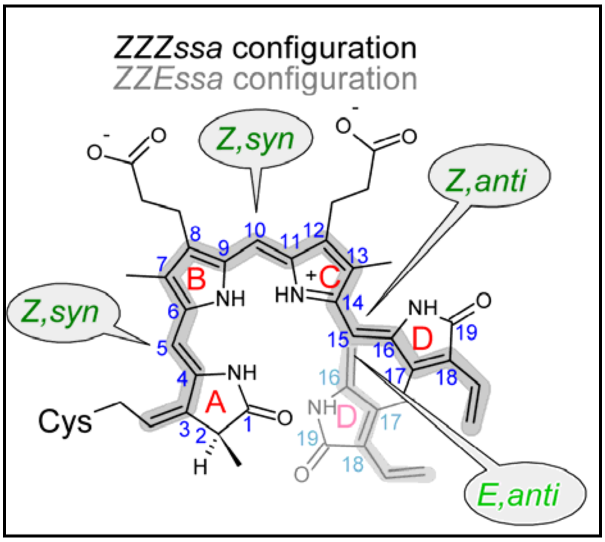

The geometry of the biliverdin chromophore is denoted according to the configuration (Z/E; *Z* - "zusammen", *E* - "entgegen") and conformation (s/a; *s* - syn, *a* - anti) of the methine bridges between the pyrrole rings *A*, *B*, *C*, and *D*. In the Pr ground state, the configuration is *ZZZssa*, in the Pfr state *ZZEssa*. In the protein, the cofactor is covalently attached to a cysteine via the ethylidene substituent at ring *A*. The C atoms in rings *A*–*D* are numbered for reference. Photon absorption leads to a photoinduced *Z/E* isomerization of the chromophore around the methine bridge between rings *C* and *D*. The figure was modified based on Fig. 1a from (Nieder et al., 2011).

Nieder, J.B., Stojkovic, E.A., Moffat, K., Forest, K.T., Lamparter, T., Bittl, R., and Kennis, J.T. (2011). Pigment-protein interactions in phytochromes probed by fluorescence line narrowing spectroscopy. *J. Phys. Chem. B* 117, 14940-14950.

2. Fluorescence data analysis

The fluorescence decays were analyzed employing a Levenberg-Marquardt algorithm for the minimization of the reduced χ_r^2 after iterative reconvolution with the instrumental response function (IRF). The value of χ_r^2 depends on a parameter set $(p_1,....,p_n)$ of the chosen continuous fit function $A(t,\lambda,p_1,...,p_n)$ for the temporally discrete fluorescence points $F(t_\nu,\lambda)$. The function χ_r^2 is evaluated in each time t_ν channel (ν= 1.4096) after convolution of the determined fit function $A(t,\lambda,p_1,...,p_n)$ with the IRF and averaged over all time channels:

(S1) $$\chi_r^{\,2}(p_1,....,p_n,\lambda)=\sum_{\nu=1}^{4096}\frac{1}{\sqrt{F(t_\nu,\lambda)}}\left(\frac{F(t_\nu,\lambda)-A(t_\nu,\lambda,p_1,...,p_n)}{\sqrt{F(t_\nu,\lambda)}}\right)^2$$

$A(t,\lambda,p_1,...,p_n)$ was chosen as a triexponential decay function

(S2) $$A(t,\lambda)=\sum_{j=1}^{3}a_j(\lambda)\,e^{-t/\tau_j},$$

with the parameters $a_j(\lambda)$ and τ_j denoting wavelength-dependent amplitude $a_j(\lambda)$ and time constant τ_j of the jth exponential decay component for two components (n = 3). The triexponential fits of all decay curves measured in one time- and wavelength resolved fluorescence spectrum were performed as global fits with common values of lifetimes τ_j (linked parameters) for all decay curves and wavelength-dependent pre-exponential factors $a_j(\lambda)$ (non-linked parameters). The result of this analysis is usually plotted as a graph of $a_j(\lambda)$ for all wavelength independent lifetimes τ_j representing so-called decay associated spectra (DAS) thus revealing the energetic position of individual decay components.

The quality of the fit was judged by the value of χ_r^2 and by the degree of randomness of residuals (difference between the experimental data points and the fit at each time point t_ν) to check for the absence of any correlation of the deviations in a certain time interval. For this judgment, the autocorrelation function of the residuals was calculated, which was around 1.0 for the whole time interval. For these calculations, the software of Globals Unlimited® (University of Illinois, Urbana, USA) was used. It was found that a sufficient quality of the fit could be achieved for fitting with n=3 decay components (χ_r^2 = 1.05). No significant improvement was obtained with n>3 decay components.

2. Absorption spectra and photoconversion

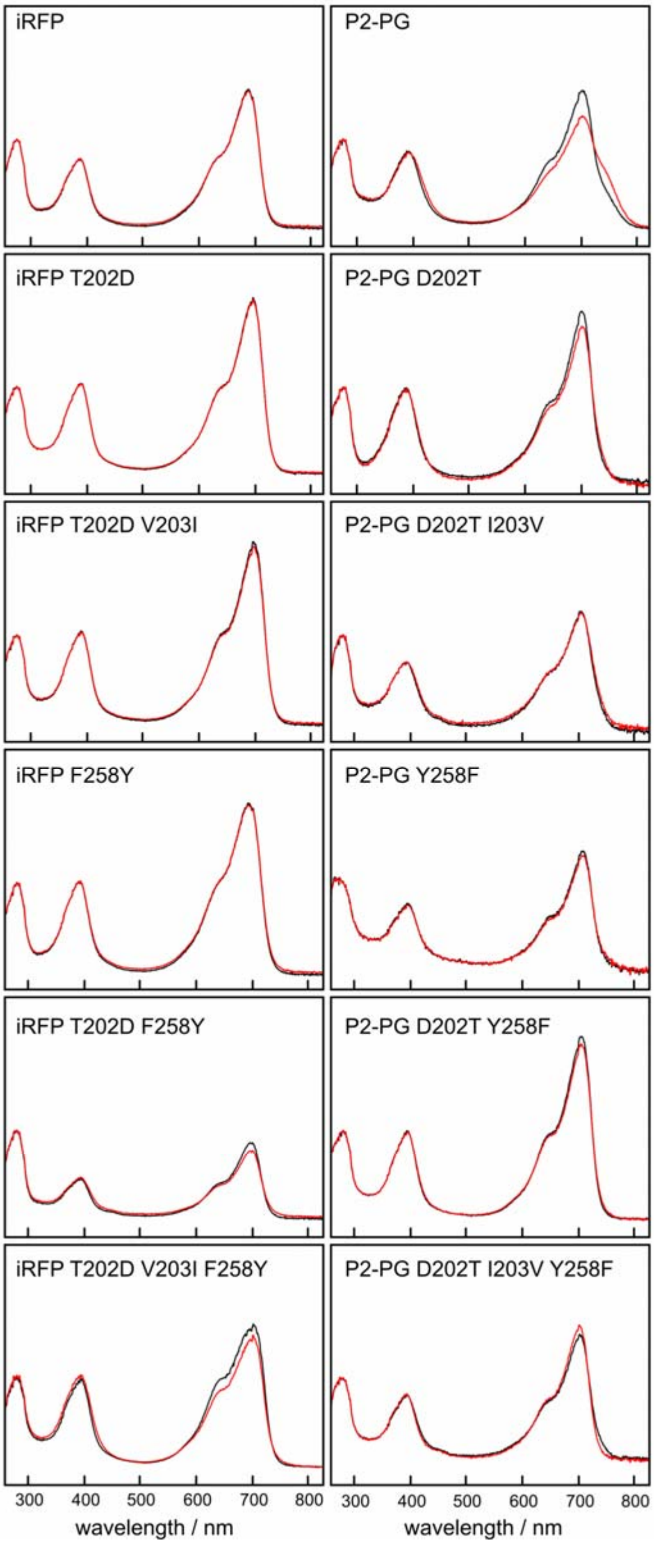

Figure S1. Normalized absorption spectra of P2PG, iRFP, and the mutants obtained via route A (right) and route B (left) showing the typical Soret (around 400 nm) and Q bands (around 700 nm) due to absorption of the biliverdin chromophore. Spectra measured before and after red-light irradiation (660 nm LED) are shown in black and right, respectively. Note that the photoactive variants only undergo a phototransformation to the Meta-R state which, due to the low extent of photoconversion, is typically reflected only by a decrease of the Q-band absorption. All spectra were normalized to the protein absorption peak at 280 nm.

3. **Fluorescence spectra**

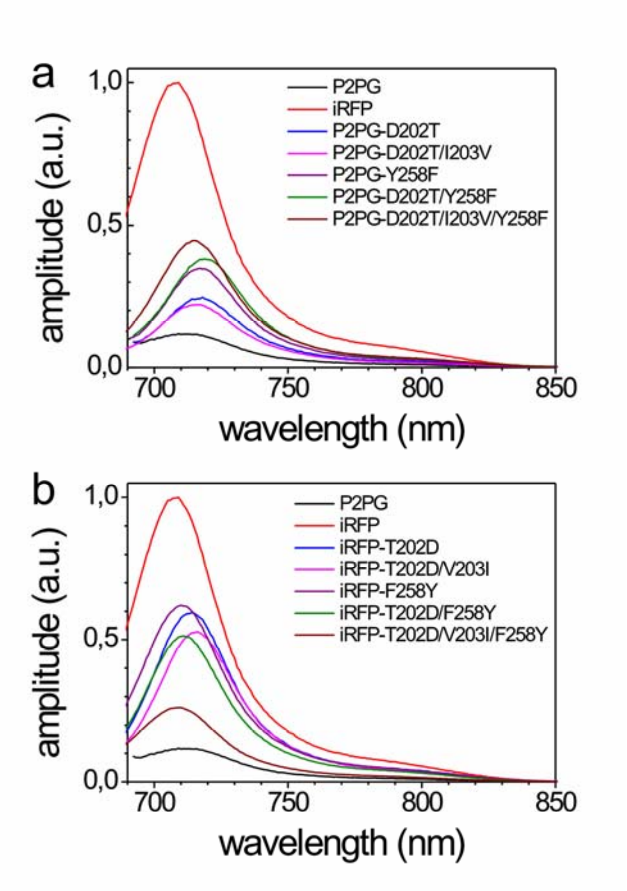

Figure S2. Fluorescence spectra measured for the various mutants, obtained via route A (left) and route B (right). For normalization, the fluorescence spectra were corrected according to the number of absorbed photons at the excitation wavelength according to the recorded UV/Vis absorption spectra using as a reference the published extinction coefficient of iRFP (85,000 M^{-1} cm^{-1}) from ref. 11. Standard solutions of the dyes Atto 680 (Attotech) in H_2O and Nile Blue (Sigma Aldrich) in ethanol (Φ_{fl} = 0.3/0.27) were used as references to determine the iRFP quantum yield (Φ_{fl} = 0.059). Subsequently, iRFP was used as a reference for determining the fluorescence quantum yields of the other phytochrome variants.

4. RR spectra in the low frequency region

Below 700 cm^{-1} there are three medium intense Raman bands which may be assigned in analogy to previous vibrational analysis of the BV chromophore in the Pfr state of Agp2 (see manuscript). In that case, theoretical calculations predicted modes of considerable Raman intensity in this region that involve torsional and out-of-plane deformation coordinates of rings D, C, and ring B, as well as the N-H out-of-plane coordinate of ring B. Guided by the predicted Raman intensities and frequencies, we assign the peak at 673 cm^{-1} (674 cm^{-1}) of iRFP (P2-PG) to two closely spaced modes involving mainly torsional and out-of-plane deformation coordinates of rings C and D (Figure S3). The 651 cm^{-1} band in the spectrum of iRFP (656 cm^{-1} for P2-PG) displays a small upshift upon H/D exchange which can be rationalized in terms of the involvement of the N-H out-of-plane coordinates in H_2O but not in D_2O. An even larger contribution of this coordinate is likely to be the origin for the disappearance of the 659 cm^{-1} band of iRFP (663 cm^{-1} in P2-PG) in D_2O, where a new band at distinctly higher frequencies (690 cm^{-1}) is detected instead. We therefore tentatively assign the 659-cm^{-1} band (iRFP) to a mode of significant N-H out-of-plane character, presumably localized at ring B, and the 651-cm^{-1} band to a torsional and out-of-plane deformation mode involving ring D (*vide supra*).

Inspection of the spectral changes brought about by the substitutions according to route A and B shows that the upshift of the HOOP mode from 810 cm^{-1} (P2-PG) to 813 cm^{-1} (iRFP) is produced by the single mutations at either the position 202 (D202T) or 258 (Y258F) (Figure S4). The latter replacement is also responsible for the intensity increase of the 663 cm^{-1} band whereas the frequency shift of this band largely depend on the remote amino acid substitutions.

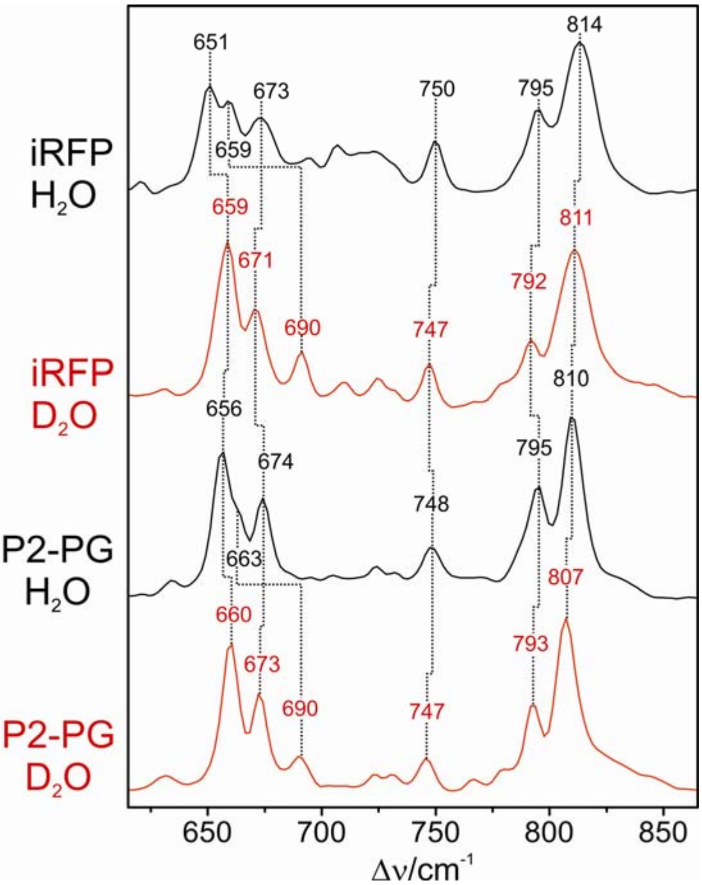

Figure S3. RR spectra of P2PG and iRFP in the HOOP region, measured from samples in H_2O (black) and D_2O (red).

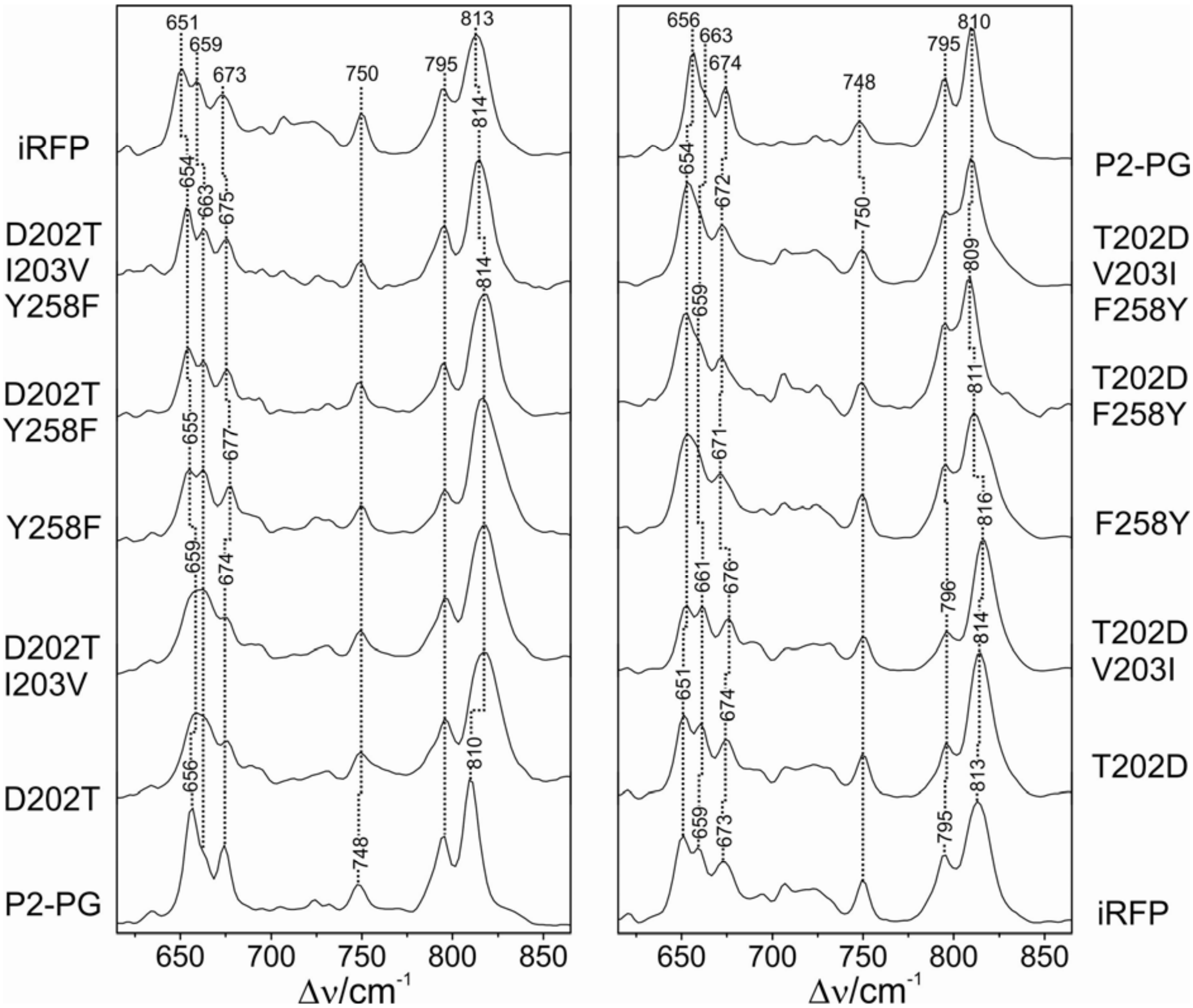

Figure S4. RR spectra of the P2-PG variants obtained via route A (left) and route B (right), compared with the spectra of the wild-type P2-PG and iRFP. The spectra, measured from the proteins in H_2O, display the region of the HOOP modes.

5. IR difference spectra

Infrared difference spectroscopy

IR difference spectroscopy can be employed only to those variants which undergo a photoisomerisation (Figure S5). Due to the lack of the PHY domain, the spectra of these variants do not display any major difference signals in the amide I and amide II region. However, the C=O stretching mode of ring *D,* which in the Pr state of P2PG is observed at 1711 cm^{-1}, can clearly be identified in all photoactive variants, albeit at slightly higher frequencies. The corresponding ring *A* C=O stretching at 1737 cm^{-1} (P2PG) can barely be detected suggesting that the position of this mode remains largely unchanged upon photoconversion.

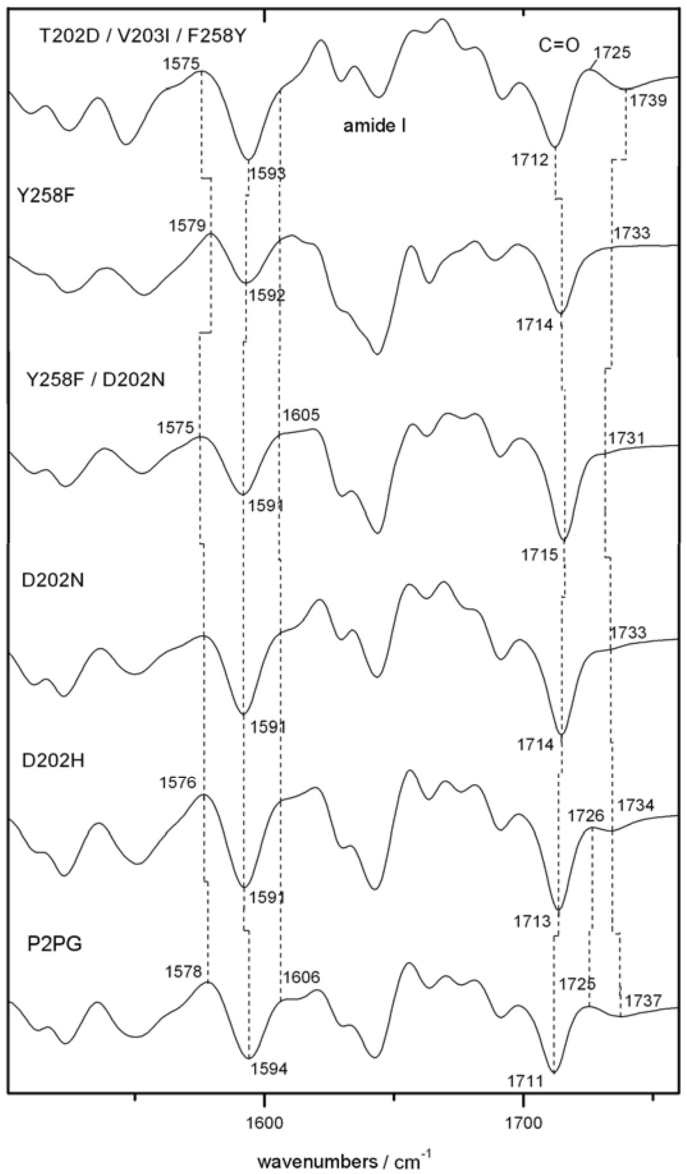

Figure S5. IR difference spectra obtained by subtracting the spectrum of the parent Pr state from that of the photoproduct obtained by 660 nm LED irradiation. Thus, negative and positive bands refer to the parent state and the photoproduct (usually a Meta-R state), respectively. The signals of the ring D and A carbonyl functions are observed between 1710 and 1725 cm^{-1} and above 1730 cm^{-1}, respectively. As expected, no major signals are observed in the amide I region.

6. RR spectra of further mutants

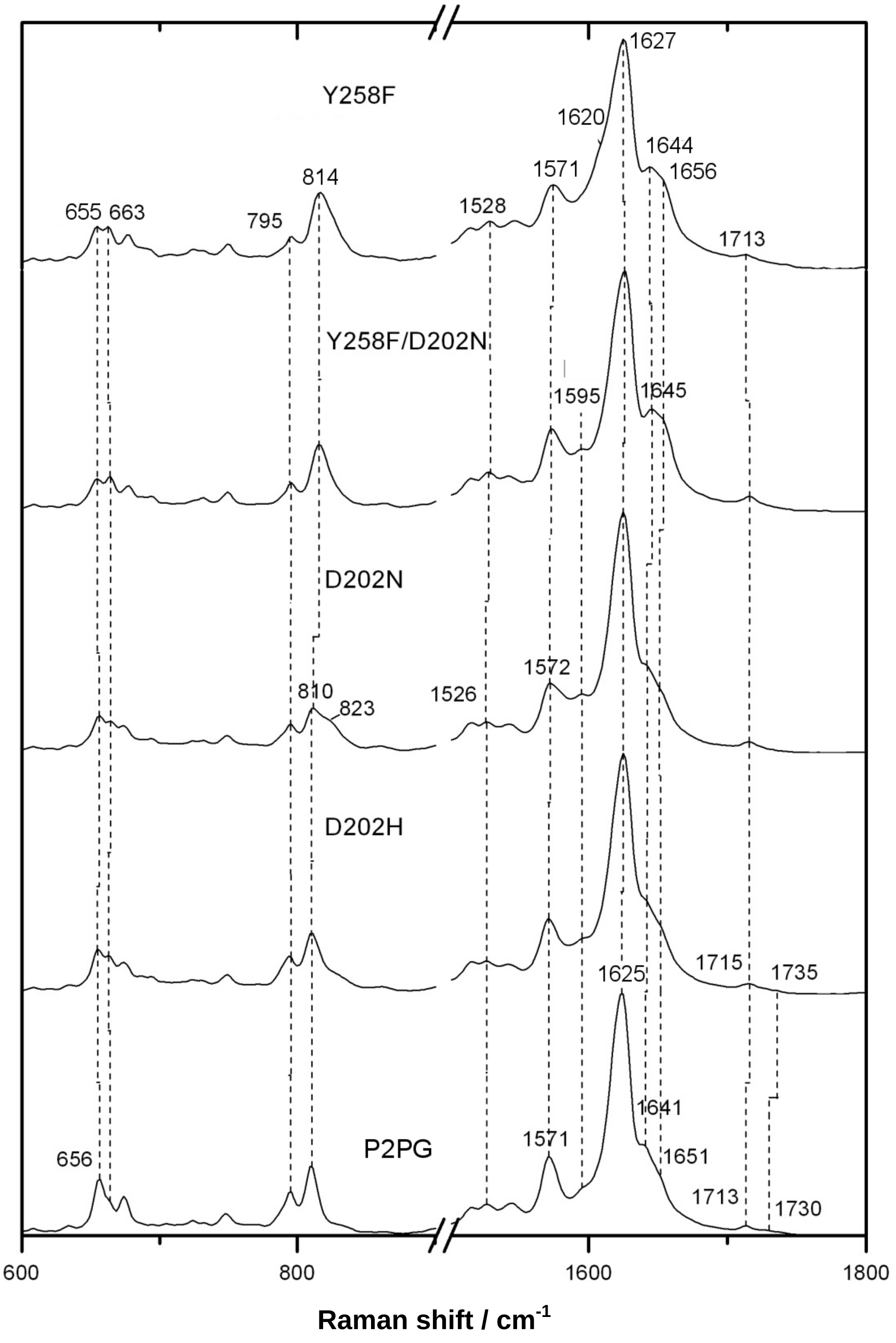

Figure S6. RR spectra of various mutants derived from P2PG.

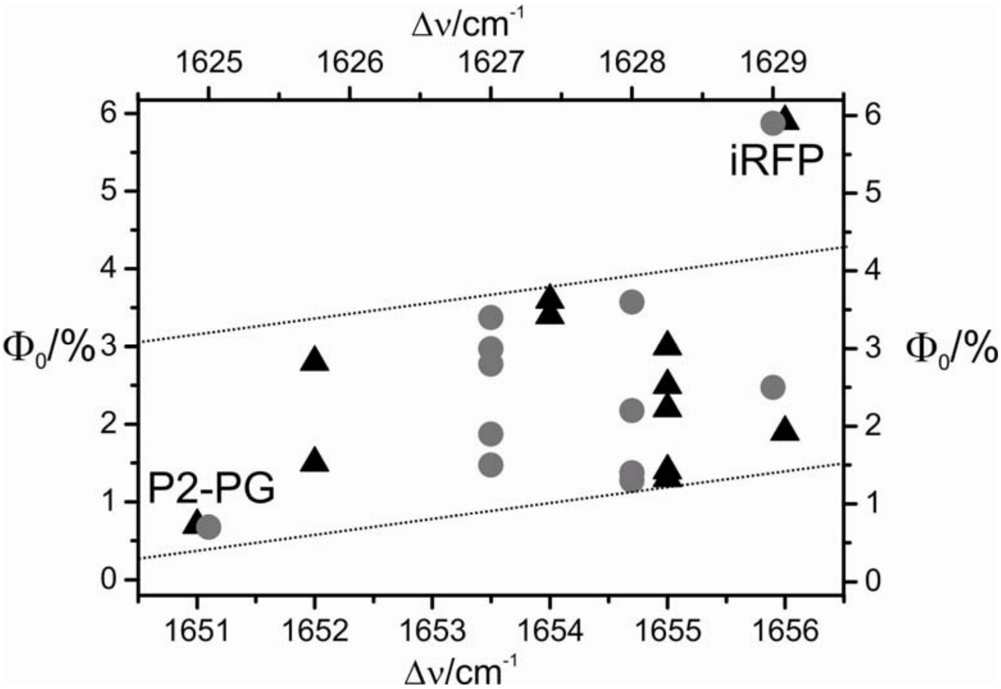

Figure S7. Correlation between the C=C stretching frequency of ring *D* (grey circles, upper abscissa) and the *A*-*B* methine bridge (high frequency component, black triangle, lower abscissa) and the experimentally determined fluorescence quantum yield Φ_{fl}.
